# Dynamic cellular biomechanics in responses to chemotherapeutic drug in hypoxia probed by atomic force spectroscopy

**DOI:** 10.18632/oncotarget.27974

**Published:** 2021-06-08

**Authors:** Lina Alhalhooly, Babak Mamnoon, Jiha Kim, Sanku Mallik, Yongki Choi

**Affiliations:** ^1^Department of Physics, North Dakota State University, Fargo, North Dakota 58108, United States; ^2^Pharmaceutical Sciences, North Dakota State University, Fargo, North Dakota 58108, United States; ^3^Department of Biological Sciences, North Dakota State University, Fargo, North Dakota 58108, United States; ^4^Cellular and Molecular Biology Program, North Dakota State University, Fargo, North Dakota 58108, United States; ^5^Materials and Nanotechnology Program, North Dakota State University, Fargo, North Dakota 58108, United States

**Keywords:** cellular stiffness, roughness, adhesion, drug resistance, hypoxia

## Abstract

The changes in cellular structure play an important role in cancer cell development, progression, and metastasis. By exploiting single-cell, force spectroscopy methods, we probed biophysical and biomechanical kinetics (stiffness, morphology, roughness, adhesion) of brain, breast, prostate, and pancreatic cancer cells with standard chemotherapeutic drugs in normoxia and hypoxia over 12–24 hours. After exposure to the drugs, we found that brain, breast, and pancreatic cancer cells became approximately 55–75% less stiff, while prostate cancer cells became more stiff, due to either drug-induced disruption or reinforcement of cytoskeletal structure. However, the rate of the stiffness change decreased up to 2-folds in hypoxia, suggesting a correlation between cellular stiffness and drug resistance of cancer cells in hypoxic tumor microenvironment. Also, we observed significant changes in the cell body height, surface roughness, and cytoadhesion of cancer cells after exposure to drugs, which followed the trend of stiffness. Our results show that a degree of chemotherapeutic drug effects on biomechanical and biophysical properties of cancer cells is distinguishable in normoxia and hypoxia, which are correlated with alteration of cytoskeletal structure and integrity during drug-induced apoptotic process.

## INTRODUCTION

Cell surface plays important roles in fundamental cellular functions such as signaling, communication, adhesion, transport, and tumor metastasis [1–4]. The cell surfaces dynamically interact with physical, chemical, and biological environments surrounding cells and thus, alteration in cell’s surface structure substantially influences overall cell functions [1, 5, 6]. In particular, deformability of cells associated with cell shape, motility, and invasion has shown implications for cell death and cancer metastasis [7, 8], which is critical information for developing new anticancer drugs with increased efficacy in cancer chemotherapy [9, 10].

Chemotherapeutics rely on the release of anticancer drugs at tumor sites and the anticancer drug-induced cancer cell death, which has been well-understood biochemically [7]. While a number of studies have shown the relationship between chemotherapy-induced cell death [7, 11] and alteration in cellular mechanics such as stiffness [8], the impact of drugs on biomechanical and biophysical properties of cancer cells is not fully understood yet. Furthermore, stiffness at the tissue-level is significantly affected by the tumor stage, invasiveness, and location within the tumor due to the deposition of extracellular matrix, which influences the cellular behavior and metastatic capacity at the single-cell level as well [12–15]. Nevertheless, cancer cells at the metastatic sites or during epithelial mesenchymal transition (EMT) have become softer and more deformable though substantial rearrangements in the cytoskeleton [16, 17].

One of the primary drivers of EMT and metastasis is hypoxia, which can induce cytoskeletal injury and remodeling through the activation of the RhoA/ROCK signaling pathway by hypoxia-inducible factor-1α [12, 18, 19]. Using breast tumors, Plodinec et al. has shown the correlation between hypoxia and the softness of cancer cells [12]. However, it is unclear whether hypoxia is the solely responsible for cancer cell softening without taking into account the surrounding tumor microenvironment. Hypoxia is also known to be involved in drug resistance through changes in cellular metabolism, drug detoxification efficiency, and genetic instability [20].

Thus, further information on dynamics of cellular elasticity, morphology, and adhesion, and correlation between them following exposure to the drugs is a key to expanding our knowledge of the drug effects on cancer cell physiology and enhancing the chemotherapeutic potential of drugs [21]. Furthermore, investigation of hypoxia on the efficacy of chemotherapeutic drugs is of major interest among biological and biomedical fields because hypoxic condition appeared in almost all solid tumors and increases the cancer cell survival and resistance to chemotherapy, leading to poor clinical outcomes [22, 23].

A variety of biomechanical and biophysical assay approaches such as micropipette aspiration [24], optical and magnetic tweezers [25, 26], mechanical microplate stretcher [27], and atomic force microscopy (AFM) [28] have been used to assess the deformability of living cells. Among those, the AFM method has proven to be an ideal technique for investigating nanoscale-resolution morphology and biomechanical properties of single cells in physiological solutions [1, 29, 30]. Furthermore, the functionalization of the AFM probe with selective ligands permits quantitative measurements of the structure and function of the intracellular components such as cytoskeleton, adhesion force and binding probability between membrane receptors and ligands [21]. Recently, the stiffness analysis of live metastatic cancer cells using the AFM method has demonstrated the applicability in distinguishing cancerous cells from normal ones [8]. Several studies using the AFM-based force measurements also have shown a significant change in cell stiffness with increasing metastatic efficiency in human cancer cell lines and chemotherapy exposure in leukemia cells [7].

In this work, we quantified the drug effects on the biomechanical and biophysical properties of four cancer cell lines: MDA-MB-231 triple negative breast cancer, PANC-1 pancreatic cancer, PC-3 prostate cancer, and U-118 MG glioblastoma cell lines. The AFM force techniques were applied to trace time-dependent changes in cellular morphology, elasticity, roughness, and adhesion after exposure to standard chemotherapeutic drugs for each of the cancer cells: gemcitabine for PANC-1, doxorubicin for MDA-MB-231, vincristine for U-118 MG, and mitoxantrone for PC-3. Comparison of such parameters in normoxia and hypoxia provides a fundamental understanding of the drug effect on cellular cytoskeletal structure and integrity and its dependence on the oxygen condition surrounding the microenvironment.

## RESULTS AND DISCUSSION

**Figure 1:**
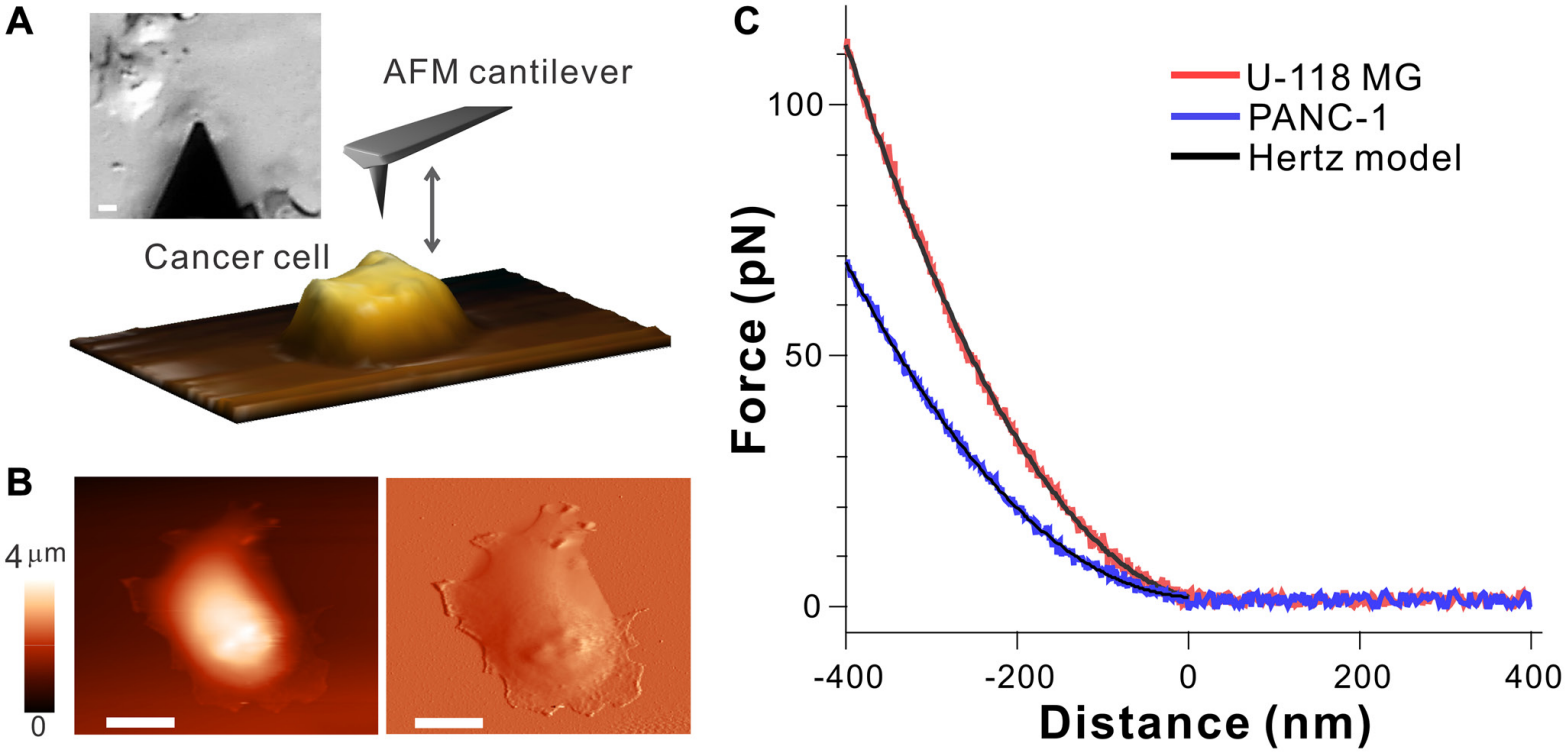
Atomic force spectroscopy probing biomechanical properties of cell surface. (**A**) Optical image and scheme of AFM cantilever and cancer cell, where the cantilever positioned above cell is brought into contact with cell surface for imaging and probing elastic modulus. The scale bar is 10 μm. (**B**) An example topographical image (left) and deflection image (right) of live PANC-1 cell. The scale bar is 10 μm. (**C**) Typical force-distance curves recorded during approaching process between the cantilever and cell surface of U-118 MG (red) and PANC-1 (blue) cells, along with the Hertz model (black), which allows to determine Young’s modulus *E*.

A schematic diagram of AFM-based stiffness measurements of cancer cells is depicted in Figure 1A in which an AFM cantilever tip is brought into contact with cell surface to probe the relative elastic response (Young’s modulus, *E*) of individual cells. To align the tip and cell surface, the tip was roughly positioned above the cell via an optical microscope, and then performed the raster scanning for imaging, where both topographical and deflection images were recorded (Figure 1B and Supplementary Figure 1). From the acquired images, the position of the tip is placed above the central cytoplasmic region of cell surface. Then, the tip approaches the cell, makes contact with the cell surface, further indents the cell surface up to 400 nm, and finally retracts from contact with the cell surface. During these processes, the deflection of the cantilever tip is recorded as a function of distance between the tip and cell surface, which finally converts to the force-distance curves (Figure 1C) using Hooke’s law (see Materials and Methods for more details).

The force-distance curves display the direct interaction between the tip and cell surface, which allows us to compare the elastic responses of each cell. Compared to the force-distance curve of PANC-1 cell, more force is required for U-118 MG cells to indent at the same depth (*d* < 0 nm), implying that the cell surface of U-118 MG is relatively stiffer than PANC-1. By fitting the non-linear region of the force-distance curve to the Hertz model (black curves in Figure 1C), the relative cell stiffness (Young’s modulus, *E*) of individual cells was calculated (see Materials and Methods for more details) [8, 31, 32]. Given inhomogeneity of cells, the force-distance curves and the Young’s modulus were collected by taking multiple indentation measurements at different locations over the central cytoplasmic regions of individual cells at the same indentation depth (400 nm).

**Figure 2:**
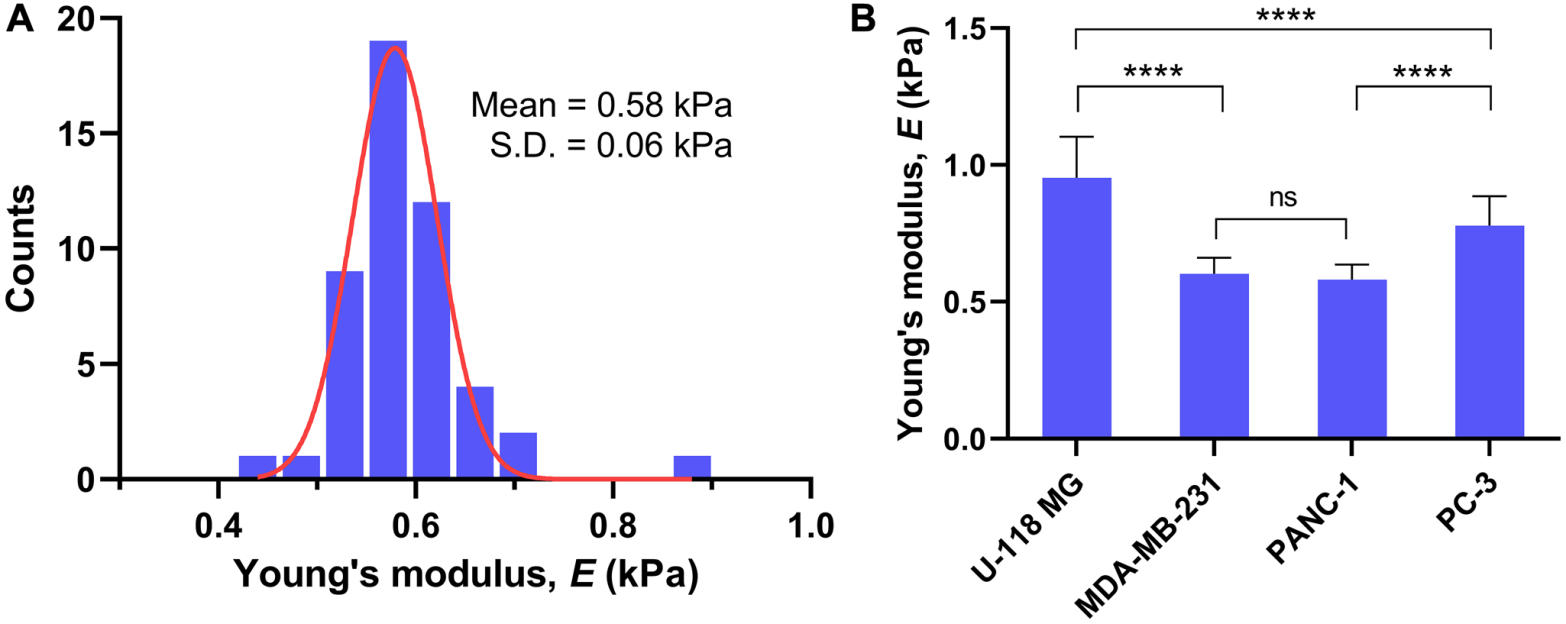
Young’s modulus E for live cancer cells. (**A**) Histogram of *E* for PANC-1 cells (*n* = 49 cells; 7 force-distance measurements per cell) pre-incubated in normoxia for 48 hours and its fit to Gaussian distribution. (**B**) Comparison of Young’s modulus *E* for four different cancer cells (U-118 MG, *n* = 9; MDA-MB-231, *n* = 10; PANC-1, *n* = 49; PC-3, *n* = 10; 7 force-distance measurements per cell) pre-incubated in normoxia for 48 hours. Data are mean ± s.d., one-way analysis of variance (ANOVA), post-hoc Tukey test; ns, not significant; ^****^ *P* < 0.0001.

To evaluate distribution of the cell elasticity, a histogram of the Young’s modulus was generated from 49 live PANC-1 cells, where 7 force-distance curves per cell were measured (Figure 2A). The histogram fits well to a Gaussian distribution, determining the mean *E* of 0.58 kPa with a standard deviation (s. d.) of 0.06 kPa. Figure 2B compares the stiffness of four cancer cells pre-cultured in the media for 48 hours under normal condition (normoxia). The mean *E* of the cancer cells examined in this work ranged from 0.58 to 0.95 kPa, which agrees with previously reported *E* values of the cancer cells [33–36]. Such low *E* values across cancer cells indicate that the cells are easily deformable, potentially increasing adaptiveness to the environment and metastatic capacity. The mean *E* values (mean ± s. d.) for U-118 MG, MDA-MB-231, PANC-1, and PC-3 cells were 0.95 ± 0.15 kPa, 0.61 ± 0.06 kPa, 0.58 ± 0.06 kPa, and 0.78 ± 0.11 kPa, respectively. These results suggest that U-118 MG and PC-3 cells are relatively stiffer than MDA-MB-231 and PANC-1 cells, while the stiffness of MDA-MB-231 and PANC-1 cells revealed no statistical differences (*P* > 0.05).

**Figure 3:**
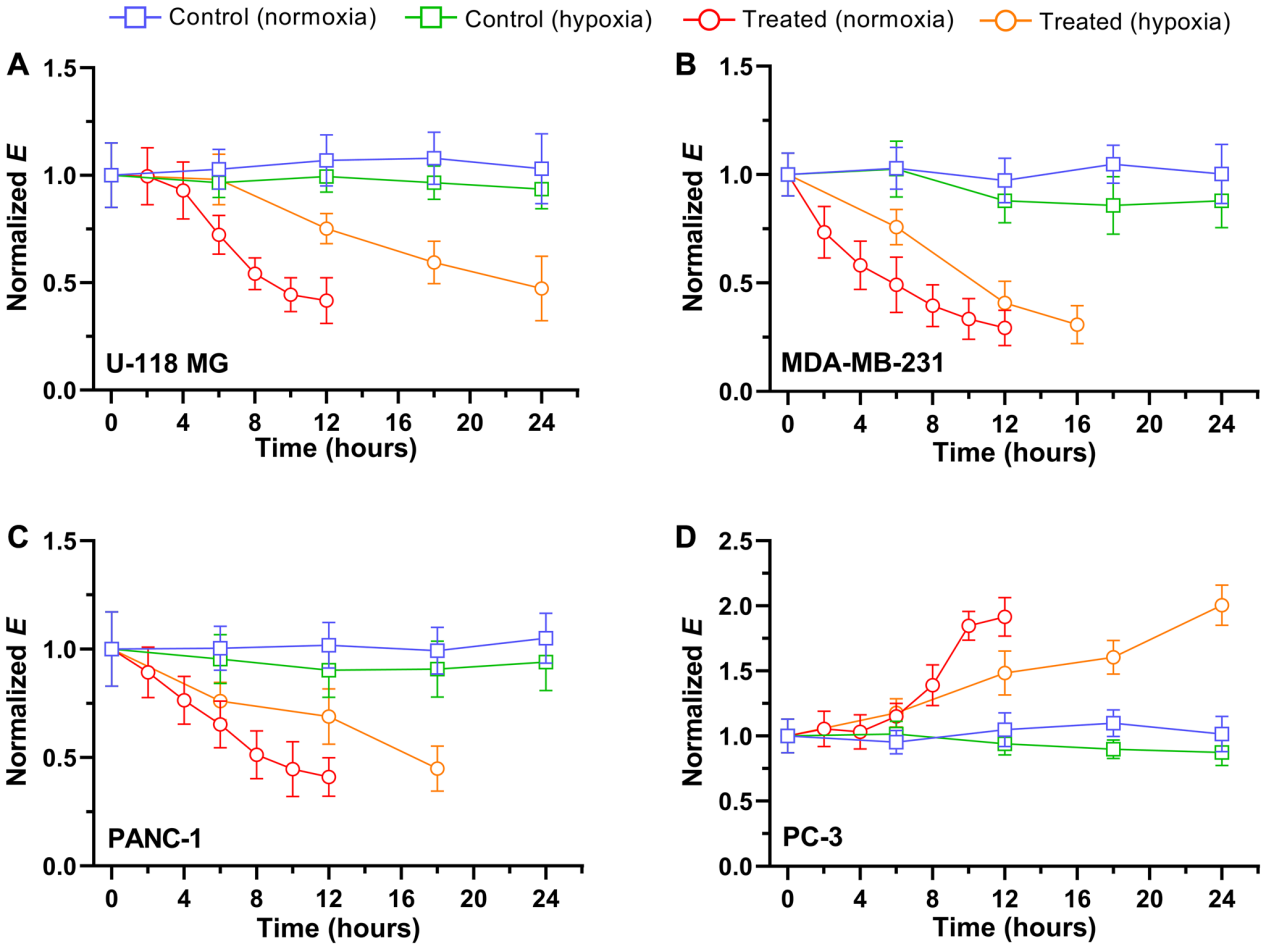
Time trace of normalized Young’s modulus E after exposure to 5 μM drugs in normoxia and hypoxia. The blue and green square represent the normalized *E* of untreated, control cells of (**A**) U118 MG, (**B**) MDA-MB-231, (**C**) PANC-1, and (**D**) PC-3 in normoxia (*n* = 5 per cell line) and hypoxia (*n* = 5 per cell line), respectively. The red and orange represent the normalized *E* of (A) U-118 MG cell exposed to vincristine, (B) MDA-MB-231 cell exposed to doxorubicin, (C) PANC-1 exposed to gemcitabine, and (D) PC-3 exposed to mitoxantrone in normoxia (*n* = 5 per cell line) and hypoxia (*n* = 5 per cell line), respectively. Data are mean ± s.d.

After initial assessments of cell stiffness, stiffness kinetics following exposure to chemotherapeutic drugs were examined under two oxygen abundancy-dependent conditions: normoxia and hypoxia (Figure 3). The optimal dose and time of drug treatment associated with a change in the cell’s stiffness was determined by our dose-response curves with viability for each cell line (Supplementary Figure 2). The measured *E_t_* values for each time point were converted to a normalized *E* (= *E_t_*/*E_t_*_=0_) using the *E_t_*_=0_ values in Figure 2B, in order to compare relative changes in *E*. Each cancer cell was pre-cultured in the media for 48 hours under normoxic condition prior to time-dependent experiments. In control measurements performed in normoxia (Figure 3A, blue curve) and hypoxia (Figure 3A, green curve), the stiffness of U-118 MG cells exhibited little or no changes in both conditions during 24 hours of measurements. The linear regression analysis to both measurements revealed that trends of *E* values were not significantly different (*P* > 0.05). These observations indicate no significant changes in the cell stiffness during 24 hours of additional culture in hypoxic condition after 48 hours of pre-culture in normoxia. Considering the inherent hypoxic tumor microenvironment of glioblastoma [37], cancer cells are expected to be adapted to the hypoxic culture condition, and thus, no significant changes were recorded in biomechanical properties.

Following exposure to the chemotherapeutic drug vincristine, the stiffness of U-118 MG cells was significantly decreased in a time-dependent manner for both normoxia and hypoxia. The *E* value began to slowly decrease within the first 4 hours of exposure, and then rapidly dropped to ~42% of the initial *E* value until 10–12 hours of exposure in normoxia (Figure 3A, red curve). After 12 hours of drug treatment, the mean *E* and one standard deviation were 0.40 and 0.13 kPa, respectively. The majority of treated cells underwent deformation after 12 hours in normoxia, which can be seen as a small, rounded shape of cells either loosely attached on the substrate or completely detached from the substrate (Supplementary Figure 1). Also, the central cytoplasmic region of cells was not identifiable due to the changes in cellular morphology after 12 hours of treatment, further limiting the evaluation of cell stiffness beyond this time point. While a similar trend of *E* was observed in hypoxia after vincristine treatments, the overall decrease was slower and gradual over 24 hours of exposure time (Figure 3A, orange curve). When cells were treated in hypoxia, it took twice as long to reach the lowest *E* value (~ 0.45 kPa) compared to cells treated in normoxia.

The chemotherapeutic drug vincristine is a potent microtubule-destabilizing agent and widely used to treat several types of cancers [38, 39]. The disruption of microtubules leads to reorganization of cytoskeletal structures and change in the cell integrity. Previous research has shown that, due to the depolymerization of cytoskeleton, the stiffness of several cancer cells and peripheral sensory neurons decreases and becomes more elastic after treating them with vincristine [40], which is consistent with our observation of significant changes in vincristine-treated cell stiffness. Interestingly, our data show that the stiffness changes are much slower in hypoxic condition. This result suggests that hypoxia could contribute to drug resistance by delaying the biomechanical dysregulation process induced by the drug.

To further investigate the relationship between a chemotherapeutic drug-induced change in stiffness and cancer type, three additional cancer cells were examined: breast, prostate, and pancreatic cancer cell lines. The time-traced, control measurements of stiffness for three cancer cells in normoxia and hypoxia were analogous to those obtained with U-118 MG cells (Figure 3B–3D). These results suggest hypoxia itself has no significant effect on the biomechanical structure of cancer cells. Next, each cell line was exposed to standard chemotherapeutic drugs including doxorubicin (MDA-MB-231), gemcitabine (PANC-1), and mitoxantrone (PC-3), and traced in normoxia and hypoxia by serial, single-cell stiffness measurements over 12–24 hours. In case of MDA-MB-231 and PANC-1, the *E* values were significantly decreased upon exposure to the drugs in time-dependent fashion. In normoxia, both cells became approximately 14–18% less stiff after every 2 hours of exposure and reached the lowest *E* values of 0.15–0.25 kPa within 12 hours of exposure. While the stiffness of both treated cells was reduced in a time-dependent manner in hypoxia, the decreasing rate of *E* was approximately 30–36% lower than in normoxia. Although the rates are slightly different for each cell, the overall changes in stiffness of three cancer cells (U-118 MG, MDA-MB-231, and PANC-1) in hypoxia were consistent with exposure to the chemotherapeutic drugs and their exposure time.

**Figure 4:**
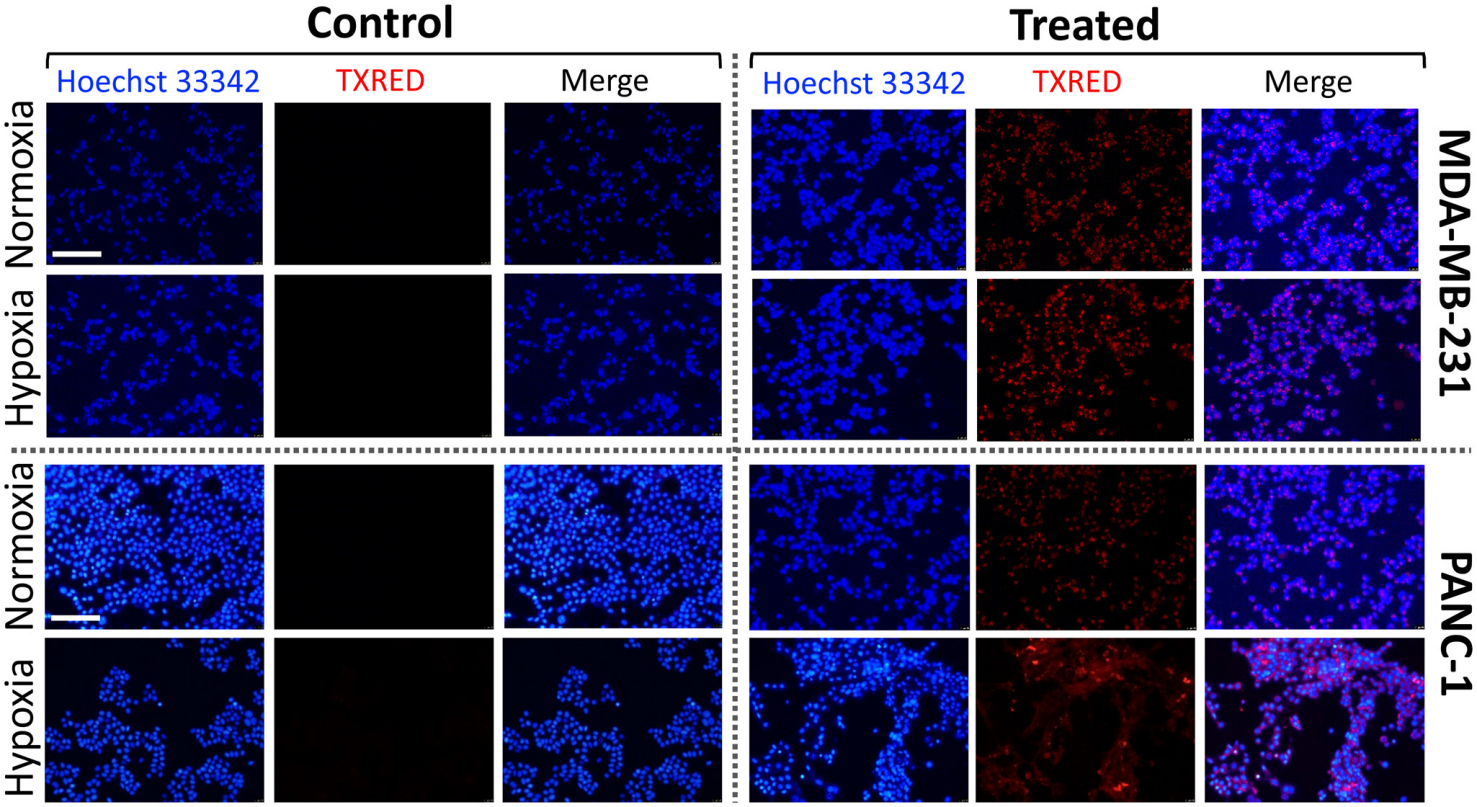
Drug uptake efficiency in normoxic and hypoxic conditions. MDA-MB-231 and PANC-1 cells were treated with doxorubicin and gemcitabine for 3 hours, respectively. Both drugs are detected by Texas Red (TXRED) filter using a fluorescence microscope. Nuclear localization of the drugs is confirmed by colocalization with Hoechst 33342. Scale bar of 100 μm is applied to all images.

Similar to vincristine, doxorubicin and gemcitabine are two of most effective chemotherapy drugs for several cancer treatments. While both drugs are well known to inhibit DNA synthesis [39, 41], a number of action mechanisms have been proposed for drug-mediated cell deaths. For example, doxorubicin was shown to intercalate nucleic acids and participate in depolymerization of actin filaments, destabilization of cytoskeletal structures, thus reducing the biomechanical strength of a cell, such as stiffness [42, 43]. A significant decrease of Young’s modulus and a change in cellular morphology of lung cancer cells treated by methotrexate, classified as an antimetabolite like gemcitabine, were also previously reported [44]. Thus, our observations of the reduced stiffness of MDA-MB-231 and PNAC-1 cells following exposure to drugs demonstrate that these types of chemotherapeutic drugs eventually induce disruption of cytoskeletal structure by either direct or indirect interaction with cytoskeletal components. Furthermore, our results from elastic measurements of all cancer cells in hypoxia indicate that the cancer cells adapt to hypoxic microenvironment and become more resistant to the drug. To rule out the possibility that attenuated cellular response to the drug in hypoxia is due to less efficient drug delivery into the cells, we investigated the amount of drug uptake by cells in normoxia and hypoxia (Figure 4). Intrinsically fluorescent doxorubicin [45, 46] and gemcitabine loaded into lissamine rhodamine nanocarriers were used for the detection of drugs using fluorescence microscopy (see “Materials and Methods” for more details) [47, 48]. After 3 hours of drug treatment, nuclear localization of drugs was observed. In both cell lines, more than 95% of the cells treated with drugs showed fluorescence localized within the nucleus (Figure 4), indicating oxygen levels did not affect the drug uptake. Considering that there was no difference in cell stiffness between normoxia and hypoxia without drug treatments, the milder stiffness change is likely due to the specific interaction of drugs with DNA and cytoskeletal components in hypoxia.

Interestingly, PC-3 cells exhibited a reversed trend: the stiffness of PC-3 cells began to increase after exposure to the chemotherapeutic drug mitoxantrone (Figure 3D). In normoxia, cell stiffness increased significantly after 6 hours of treatment and reached a two-fold increase by 12 hours. Similarly, cell stiffness increased in hypoxia in a time-dependent manner, except stiffening took nearly twice longer compared to normoxia. Note that PC-3 cells treated with mitoxantrone for 12 hours in normoxia and 24 hours in hypoxia were loosely attached on the substrate, or completely detached from the substrate and floating, which limited further evaluation of the cell stiffness beyond this time point. While the drug treatment exerted the opposite effect on cellular stiffness in the case of PC-3 cells, hypoxia played a similar role in attenuating changes of the biomechanical property of cells upon drug treatment. Like other chemotherapeutic drugs, mitoxantrone targets an enzyme to mediate DNA damage. This drug interferes with the action of DNA topoisomerase II associated with many DNA metabolic events such as transcription and replication [49]. Despite the similarity in the action mechanism of doxorubicin and mitoxantrone, the reverse results from stiffness measurements with PC-3 cells following treatment with mitoxantrone suggest that mitoxantrone induces further polymerization of cytoskeletal filaments and thus increases cell stiffness. Similar findings have been previously reported; antitumor antibiotic topotecan treatments with several patient metastatic tumor cells led to an increase in stiffness [50], and treating leukemia cells with either dexamethasone or daunorubicin resulted in increased cell stiffness by nearly two orders of magnitude [7]. Thus, drug-induced biomechanical reinforcement could be dependent on the type of cancer cells and chemotherapeutic drugs, though the underlying mechanism is unclear.

Hypoxia induces the gene expression patterns through hypoxia-inducible transcription factors and activates the expression of numerous hypoxia-response genes including cell adhesion, extracellular matrix, and cytoskeleton [51, 52]. For example, hypoxia influences expression and activation of Rho guanosine triphosphatases, which plays an important role in the regulation of the actin cytoskeleton [53]. However, dynamics and quantification of cytoskeletal changes are complex and differ from cells to cells. Furthermore, the interplay between hypoxia and chemotherapeutic treatments remains unclear. Our observations (Figure 3) suggest that drug-induced cytotoxicity could stimulate and accelerate such hypoxic effects on cytoskeletal regulation, leading to a gradual change in the stiffness. Hypoxia itself, on the other hand, induced little change to stiffness in the absence of drugs, suggesting that either biochemical and metabolic processes associated with hypoxia could be a slow process. Otherwise, the interplay between hypoxia and microenvironmental factors including drug-induced cytotoxicity might be a key to activating and accelerating the processes. In addition, several studies have shown that tumor tissues are considerably more rigid compared to the normal tissue due to the stiffening of the peripheral tumor stroma [14, 15]. Thus, the stiffness of individual cancer cells determined by the changes of intracellular cytoskeletal structure may not be directly reflected in the overall stiffness of tridimensional tumor tissue, which relays on the alteration of the tumor stroma, rather than cancer cells [12].

**Figure 5:**
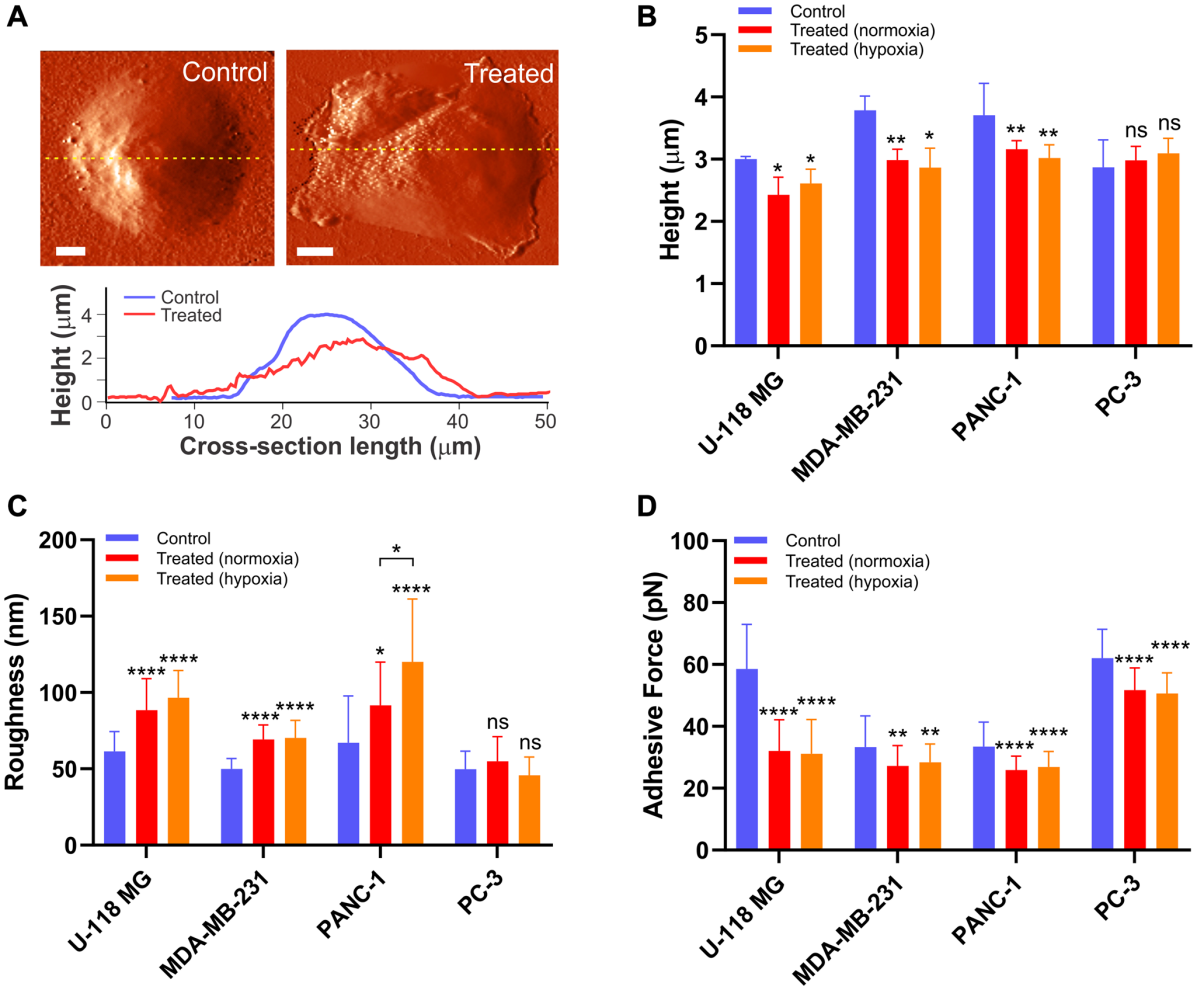
The drug-induced morphological alteration in cancer cells. (**A**) An example deflection image of live PANC-1 cell before (left) and after (right) gemcitabine treatment for 12 hours under normoxia. The scale bar is 5 μm. The cross-section analysis (yellow lines in the AFM images) shows changes in cell body height and roughness. Alteration in (**B**) apparent cell height above the cytoplasmic region (*n* = 5 per group), (**C**) surface roughness obtained from non-curved region of cell image (*n* = 5 per group), and (**D**) non-specific adhesion (*n* = 6 per group) between the AFM probe and cell surface before (blue) and after exposure to the drugs in normoxia for 12 hours (red) and hypoxia for 16–24 hours (orange). Data are mean ± s.d., Repeated measured one-way ANOVA, post-hoc Tukey test; ns, not significant; ^*^ *P* < 0.05; ^**^ *P* < 0.01; ^***^ *P* < 0.001; ^****^ *P* < 0.0001. Statistics between normoxia and hypoxia showed no statistical difference (ns) unless otherwise noted.

To examine the relationship between cell biomechanics and cell structures induced by chemotherapeutic drugs, the morphological changes of cancer cells were also investigated. Two parameters including cell body height and roughness were quantified and compared for individual cells before and after drug exposure for 12 hours in normoxia and 16–24 hours in hypoxia (Figure 5). The representative AFM images of control and treated PANC-1 cell display significant morphological changes after chemotherapeutic drug exposure (Figure 5A). First, the cross sectional analysis of AFM images along the central cytoplasmic region of the cell revealed that the cell body decreases in height after exposure to drugs for U-118 MG, PANC-1, and MDA-MB-231 cells (Figure 5B). The apparent height was reduced by 13–25% from the initial height in both normoxia and hypoxia, but the changes in cell body height were indistinguishable between normoxia and hypoxia (*P* > 0.05). These results are consistent with stiffness changes for those cells, reflecting that the alteration in cell morphology is coupled with disruption of the cytoskeleton structure induced by drug treatments. Second, the roughness of individual cells was analyzed before and after treatments (Figure 5C), which is a sensitive measure of the structure and integrity of membrane-cytoskeleton interface [54]. The root-mean-squared roughness of those cells after exposure to chemotherapeutic drugs was increased by 39–75% as compared to non-treated cells. These observations imply that the structure of the cell’s surface became less homogeneous and lost its structural integrity because of the depolymerization of cytoskeletal filaments associated with drug-induced cell death process [40, 55, 56]. Despite the substantial increase in stiffness of PC-3 cells after mitoxantrone treatments, no significant change in cell body height and roughness of cell surface was observed in neither normoxia nor hypoxia following exposure to the drug. As might be expected, the reinforcement of cytoskeletal structure does not appear to alter the apparent cell morphology.

In addition to apparent changes in cell morphology, variations of cell surface adhesion were examined to understand the relationship between cellular elasticity and adhesive force. The adhesion force spectroscopy between the AFM cantilever tip and the cell surface was recorded while retracting the tip from cell after reaching the target indentation depth of 400 nm (Supplementary Figure 3). Like morphology measurements, the three cancer cells (U-118 MG, MDA-MB-231, and PANC-1) exposed to the drugs exhibited a significant decrease in non-specific adhesion between tip and cell surface in both normoxia and hypoxia (Figure 5D). Such changes are attributed to alterations in adhesive membrane molecules associated with degradation of the cytoskeletal structure induced by the drugs [57]. Therefore, these cells ultimately lose adhesion completely and are separated from the substrate, making them more round and a balled-up shape was observed in microscopic images (Supplementary Figure 1). Notably, the adhesion analysis for PC-3 cells also exhibited reduced cellular adhesion when exposed to mitoxantrone, which could be due to down-regulation in expression of the cell signal molecules and disruption of focal adhesion during the cell death process [58]. These results suggest that adhesion is independent of type of cancer cells and drugs and it could be considered as an indicator of drug-induced apoptotic cell death.

**Figure 6:**
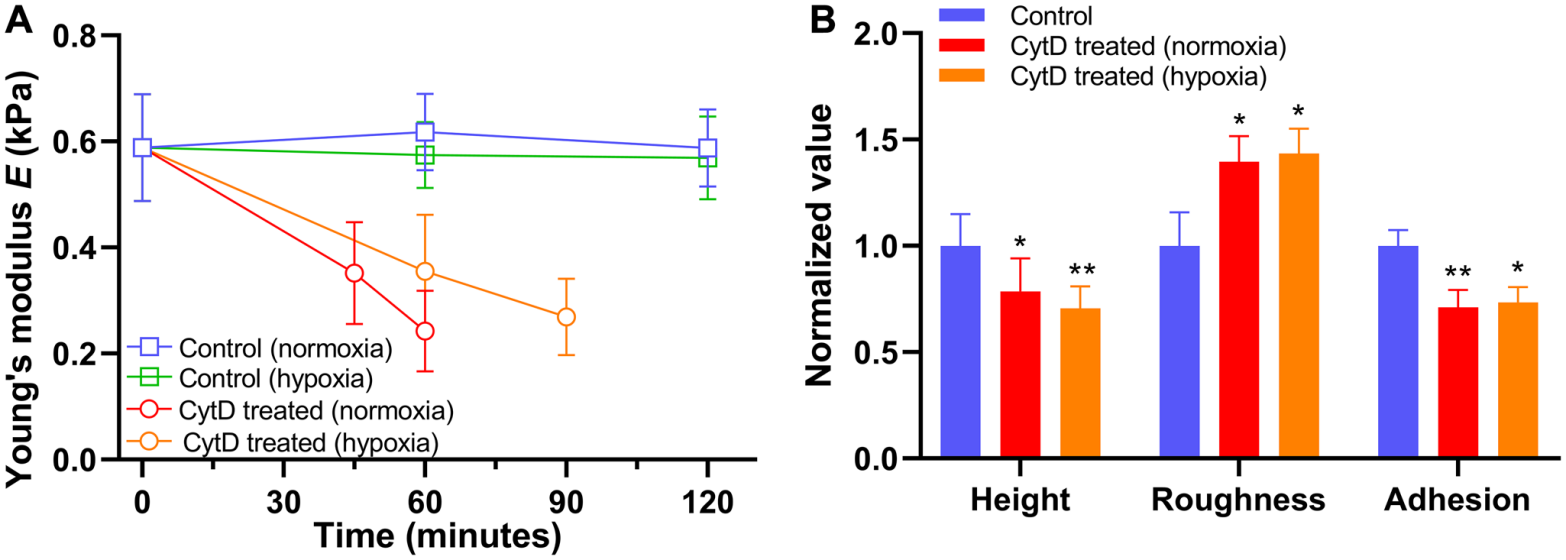
Alteration in biomechanical properties of PANC-1 cells exposed to 5 μM cytochalasin D (CytD) in normoxia and hypoxia. (**A**) Time trace of Young’s modulus *E* in normoxia (*n* = 5) and hypoxia (*n* = 5) after exposure to cytochalasin D. (**B**) Normalized values of cellular height, roughness, and adhesion measured after exposure to cytochalasin D in normoxia for 60 minutes (*n* = 5) and hypoxia for 90 minutes (*n* = 5). Data are mean ± s.d., Repeated measured one-way ANOVA, post-hoc Tukey test; ns, not significant; ^*^ *P* < 0.05; ^**^ *P* < 0.01; ^****^ *P* < 0.0001. Statistics between normoxia and hypoxia showed no statistical difference (ns).

Finally, we have examined changes in biomechanical parameters of cancer cells exposed to an inhibitor of actin polymerization cytochalasin D in normoxia and hypoxia [59]. The PANC-1 cells became less stiff under increasing duration of exposure to cytochalasin D, and the decrease of stiffness was slower in hypoxia than normoxia (Figure 6A). Also, morphology and non-specific binding force measurements of the cells exposed to cytochalasin D showed a reduction in cell height and cellular adhesion, but an increase in cellular roughness (Figure 6B). Similar changes in biomechanical properties of other cancer cell lines treated with chemotherapeutic drugs and cytochalasin D suggest that the drug-induced cytotoxicity is partly due to dynamic changes in the cytoskeletal structure (Supplementary Figure 4) [60, 61]. Also, hypoxia attenuated the cytoskeletal changes, which might contribute to drug resistance in the context of the tumor microenvironment. Although it is difficult to generalize drug effects on biomechanical and biophysical parameters of cancer cells, a combination of these parameters could help identify and distinguish the drug-induced apoptotic process in normoxia and hypoxia.

## MATERIALS AND METHODS

### Cell culture

The MDA-MB-231 triple negative breast cancer, PANC-1 pancreatic cancer, PC-3 prostate cancer, and U-118 MG glioblastoma cell lines were purchased from American Type Culture Collection (ATCC, Manassas, VA). The cells were cultured in Dulbecco’s Modified Eagle Medium (DMEM) supplemented with 10% fetal bovine serum (Avantar Seradigm) and 1% v/v antibiotics (Penicillin, Streptomycin, Amphotericin B solution, Corning). All chemotherapy drugs including doxorubicin, gemcitabine, mitoxantrone, and vincristine were purchased from Sigma Aldrich. A humidified incubator containing 5% CO_2_ and 21% Oxygen at 37°C was used for normal incubation (normoxia), while for hypoxic condition a chamber supplemented with 2% oxygen and 5% CO_2_ at 37°C was used (Biospherix C21, Parish, NY).

### Cell treatment

All cell lines were cultured in T25 cell culture flasks to reach 80–90% confluency. The cells were then trypsinized (Thermo Fisher Scientific), counted, and seeded (40,000 cells) in 35 mm glass bottom dishes (ibidi GmbH, Germany). The cells were incubated for 48 hours followed by the drug treatment. The MDA-MB-231, PANC-1, PC-3, and U-118 MG cells were treated with 5 μM doxorubicin, gemcitabine, mitoxantrone, and vincristine, respectively, for various times including 2, 4, 6, 8, 10, 12, 16, 18, and 24 hours under both hypoxic and normoxic conditions. The time intervals were determined based on the cell viability in each condition, which allows us to monitor a continuous change in biomechanical properties without missing any sudden change and determine time-dependent trends for them. At the end of treatment time points, the cells were washed with phosphate buffer saline (PBS) for 2 times and replenished with DMEM before AFM imaging and spectroscopy measurements. For cytochalasin D experiments, the PANC-1 cell line was seeded in the glass bottom dishes (40,000 cells per dish, 4 dishes for each cell line) and pre-incubated for 48 hours. The cells were then separately treated with 5 μM cytochalasin D (Cayman Chemical, Ann Arbor, MI) for 45 and 60 minutes under normoxia, and for 60 and 90 minutes under hypoxia. At the end of treatment time points, the cells were washed with PBS for 2 times and replenished with DMEM before AFM measurements.

### Fluorescence microscopy

The PANC-1 and MDA-MB-231 cells were seeded into 12-well cell culture plates (5,000 cells per well) and incubated in either normoxia or hypoxia for 48 hours. The MDA-MB-231 cells were then treated with 5 μM doxorubicin for 3 hours. Gemcitabine was loaded into nanocarriers composed of biocompatible and biodegradable polylactic acid (PLA) and polyethylene glycol (PEG) polymers, along with fluorescent lissamine-rhodamine dye, as previously described [47, 48]. Briefly, the PLA-PEG polymer and lissamine-rhodamine dye were mixed at a 95:5 molar ratio with 0.2 mg/ml gemcitabine solution. When the cells were treated with drug-loaded carriers for 3 hours, these carriers disintegrated and released gemcitabine. Due to the presence of fluorescent lissamine-rhodamine dye, the cells were identified under fluorescent microscopy. The PANC-1 cells were treated with 5 μM gemcitabine loaded into the nanocarriers for 3 hours. Subsequently, the cells were washed with PBS for three times. The nuclei of the cells were stained with Hoechst 33342 (NucBlue™, Invitrogen) dye. Then, the cells were washed again with PBS for three times and a fluorescence microscope (Leica) was used for imaging.

### Atomic force microscopy

The cell imaging and spectroscopy measurements were conducted using a commercial AFM (NT-MDT NTEGRA) with optical viewing system and V-shaped silicon nitride AFM probes with a spring constant of 0.08 N/m (Nanoworld) at room temperature. Cantilever spring was calibrated by the thermal noise fluctuation methods [62], and the deflection sensitivity of each tip was calibrated by force-distance curve measurements on the bare glass area of the petri dish. At least 5 cells at each condition were randomly selected for all imaging and other force measurements. To prevent the false measuring of already dying or dead cells due to the drug treatment, we excluded the cells that are loosely attached or floating. The scanning resolution was 256 × 256 pixels with a scan rate of 0.1–0.5 Hz, depending on the scanning areas of irregular cell size. The acquired images were flattened, if required, to eliminate the background noise and tilt from the surface using all unmasked portion of scan lines to calculate individual least-square fit polynomials for each line.

### Stiffness and adhesion measurements

The relative cell stiffness (Young’s modulus) and cell surface adhesion were extracted from force-distance (FD) curves. The FD curves were obtained on the central cytoplasmic region of cell surface. The approaching and retracting rates of probe were 1 μm/s for all measurements. To prevent cell damage and eliminate potential substrate effects, all measurements and analysis were performed with a shallow indentation depth of cells (400 nm). The Young’s modulus was determined by fitting the FD curves with the Hertz model [8, 31, 32]. First, the FD curves were converted to force-indentation curves. The force *F* is calculated from the cantilever deflection *d* and the cantilever spring constant *k* using Hook’s law (*F* = *kd*). The tip-sample separation called indentation *δ* was calculated through the difference between relative piezo displacement Δ*z* and cantilever deflection *d* (i.e., *δ* = Δ*z* - *d*). Second, the force-indentation curves in the post-contact region were fitted by the Hertz model. Depending on the tip geometry (four-sided pyramid), the Young’s modulus can be extracted using $F(\delta )=\left(\frac{E}{1-r^{2}}\frac{\tan\alpha }{\sqrt{2}}\right)\delta^{2}$, where *E* is the Young’s modulus, *r* is the Poisson’s ratio, and α is the tip face angle. The Poisson’s ratio of 0.5 for typical soft biological samples and the tip face angle of 35° were used. The FD curve measurements typically involve non-specific adhesion between the macromolecules on the cell surface and the tip. During the tip retraction from the cell surface, the detachment force (adhesion force) required to separate the tip from the macromolecules on cell surface was determined from a rupture event in sawtooth like pattern. The adhesion force was determined as difference between force values at zero-force line of the FD curve and at the negative minimum of the FD curve (Supplementary Figure 3). The mean Young’s modulus was calculated by at least total 35 FD curves at each time point, and the mean adhesion was obtained from a total of 60 adhesion measurements at each condition.

### Surface roughness analysis

The analysis of the surface roughness of cells was carried out using the Image Analysis P9 software (NT-MDT) using acquired AFM images. The root-mean-square roughness *R_rms_* was quantified, which was given by $R_{rms}=\sqrt{\frac{1}{N_{x}N_{y}}\sum_{j=1}^{N_{y}}\sum_{i=1}^{N_{x}}Z_{ij}^{2}}$, where *Z_ij_* = *Z*(*x_i_*, *y_i_*) is the height function defined over a rectangular area in the XY plane with a uniform grid of dimensions *N_x_* and *N_y_* and of steps Δ*x*, Δ*y* along the X and Y directions, respectively [43]. For each cell, at least five areas (2.5 × 2.5 μm^2^) on the central flat region were selected and analyzed. The mean roughness was calculated by a total of 125 roughness measurements at each condition.

### Statistical analysis

All data were expressed as mean ± standard deviation and analyzed using the Prism 8 (GraphPad software). The statistical significance was determined using analysis of variance followed by suitable post-hoc test. The *p*-values lower than 0.05 were considered statistically significant.

## CONCLUSIONS

In this work, we report alteration of cancer cells’ biomechanical and biophysical properties induced by the standard chemotherapeutic drugs using AFM-based, time-traced imaging and force spectroscopy measurements. The following observations provide new information about the interplay between hypoxia and chemotherapeutic drugs. We found that stiffness kinetics depends on type of the drug, exposure time to the drug, and oxygen levels in the microenvironments, while the stiffness of untreated cancer cells remain consistent in both normoxia and hypoxia. In addition, such changes in the stiffness due to either disruption or reinforcement of cytoskeletal structure induced by the drug were coupled with substantial alteration in cellular morphology, surface roughness, and cytoadhesion. Although the drug treatment alone significantly affects the cellular stiffness, the efficacy can be dampened by drug resistance due to the hypoxia, emphasizing the complex underpinning mechanisms that govern overall biomechanical and biophysical properties.

## SUPPLEMENTARY MATERIALS


