# Supplementary material for: Dynamic cellular biomechanics in responses to chemotherapeutic drug in hypoxia probed by atomic force spectroscopy
Source: Oncotarget. 2021 Jun 8;12(12):1165–77. doi: 10.18632/oncotarget.27974 (PMC8202777; doi:10.18632/oncotarget.27974)
Supplement: Supplementary file 1 [file oncotarget-12-1165-s001.pdf]

Dynamic cellular biomechanics in responses to chemotherapeutic drug in hypoxia probed by atomic force spectroscopy

SUPPLEMENTARY MATERIALS

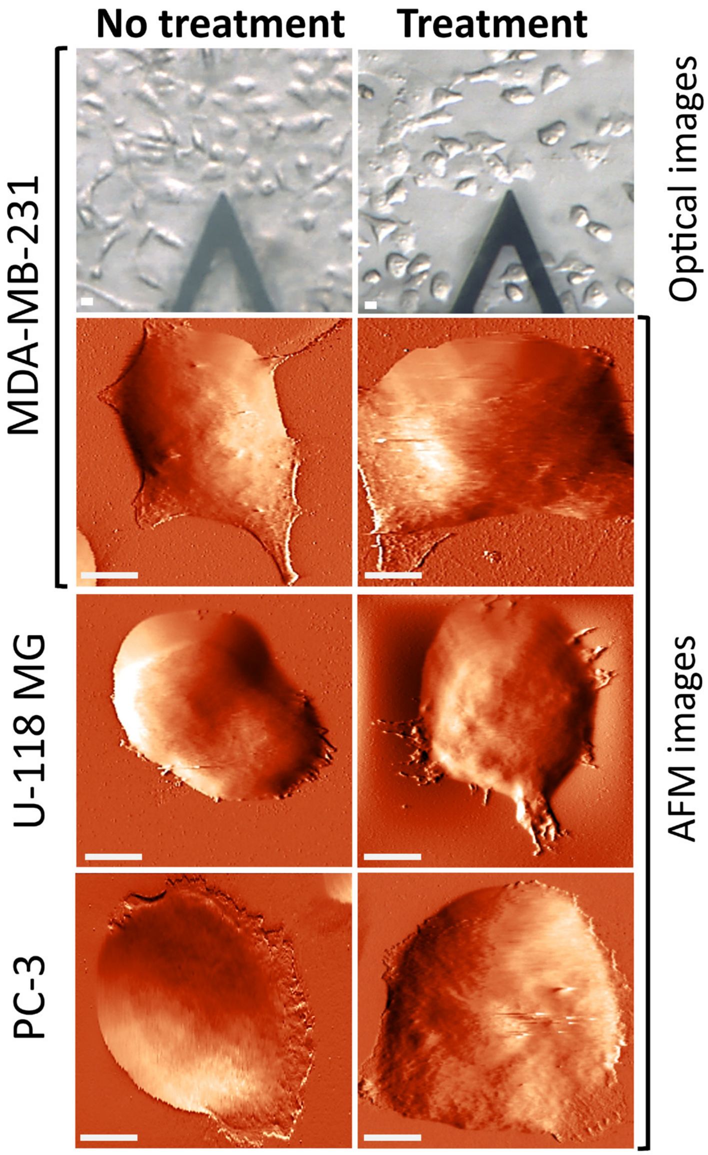

Supplementary Figure 1: Additional optical and AFM images of cancer cells before and after exposure to drug in normoxia: MDA-MB-231, U-118 MG, and PC-3 were exposed to doxorubicin, vincristine, and mitoxantrone, respectively. The scale bars in optical images and AFM images are 20 µm and 10 µm, respectively.

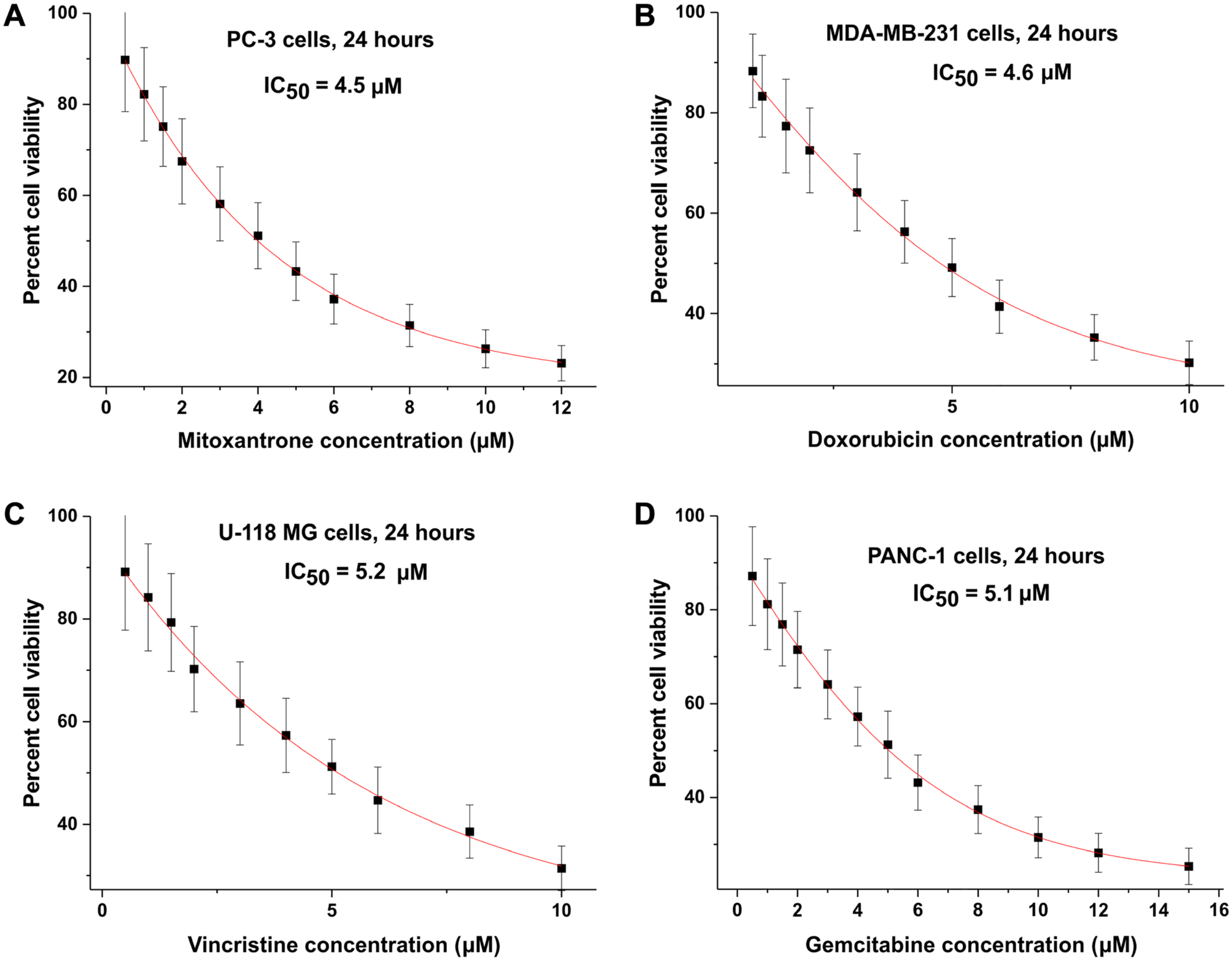

Supplementary Figure 2: Dose-response curves with viability for each cell line: (**A**) PC-3; (**B**) MDA-MB-231; (**C**) U118 MG; and (**D**) PANC-1. Each cell line was seeded in 96-well cell culture plates (5,000 cells per well) and incubated overnight. The cells were then treated with different concentrations of doxorubicin, mitoxantrone, vincristine, and gemcitabine ranging from 0.5 to 15 μM. The cells were washed 3 times with phosphate buffered saline after 12 and 24 hours. A mixture of the cell culture medium and Alamar Blue reagent (9:1 v/v ratio) was added into each well. After 5 hours, the fluorescence was measured with 560 and 595 nm excitation and emission wavelengths respectively, and the cell viability was calculated.

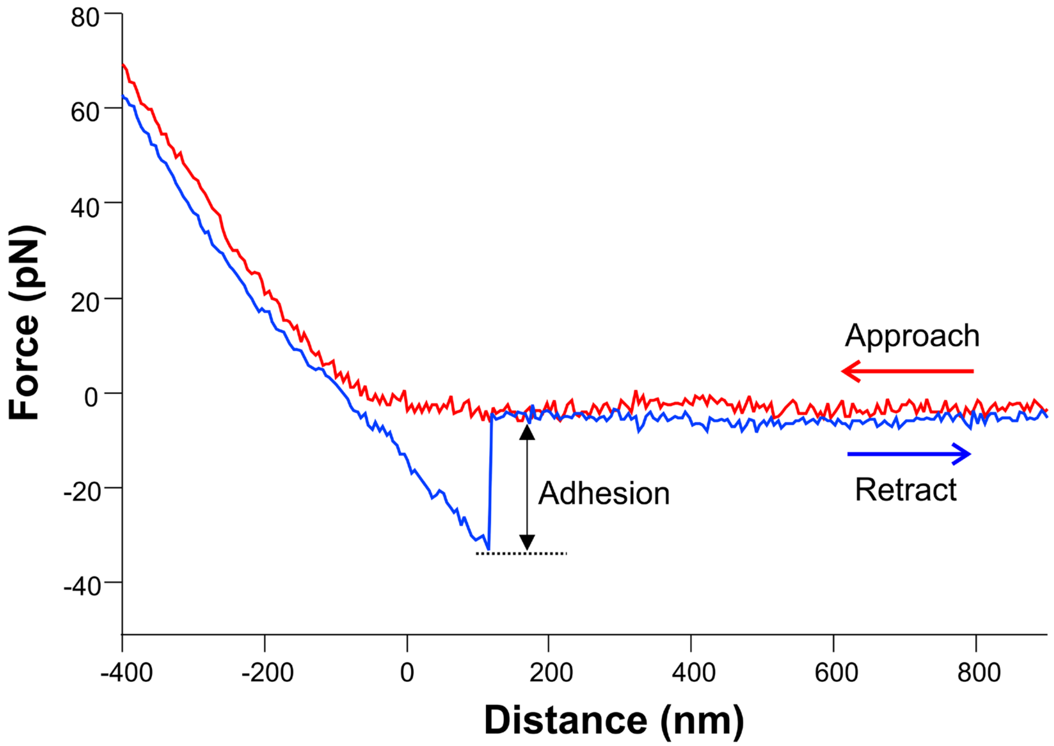

Supplementary Figure 3: An example of force-distance (FD) curve obtained from the PANC-1 cell, showing approach (red) and retract (blue) curve. When the tip is retracted from the cell surface, adhesion events take place as shown a sawtooth-like shape. The adhesion force was determined as difference between force values at zero-force line of the FD curve and at the negative minimum of the FD curve (black arrow).

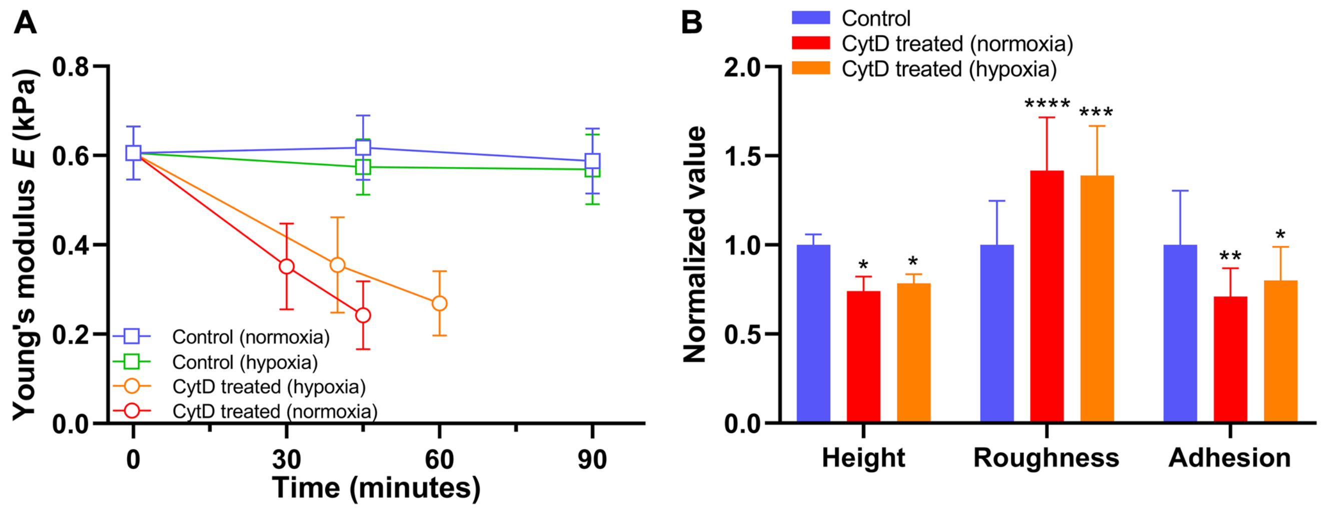

Supplementary Figure 4: Alteration in biomechanical properties of MDA-MB-231 cells exposed to 5 μM cytochalasin D (CytD) in normoxia and hypoxia. (**A**) Time trace of Young's modulus E in normoxia (*n* = 5) and hypoxia (*n* = 5) after exposure to cytochalasin D. (**B**) Normalized values of cellular height, roughness, and adhesion measured after exposure to cytochalasin D in normoxia for 60 minutes (*n* = 5) and hypoxia for 90 minutes (*n* = 5). Data are mean ± s.d., Repeated measured one-way ANOVA, post-hoc Tukey test; ns, not significant; $^{*}P < 0.05$; $^{**}P < 0.01$; $^{***}P < 0.001$; $^{****}P < 0.0001$. Statistics between normoxia and hypoxia showed no statistical difference (ns).
